# Things that go bump in the night: searching for therapeutic targets and underlying mechanisms of night-time calf cramps

**DOI:** 10.1186/1757-1146-6-S1-O14

**Published:** 2013-05-31

**Authors:** Fiona Hawke, Vivienne Chuter, Joshua Burns

**Affiliations:** 1 Podiatry Program, The University of Newcastle, Ourimbah, NSW, 2258, Australia; 2 Sydney Medical School, The University of Sydney, Westmead, NSW, 2145, Australia; 3 Arthritis and Musculoskeletal Research Group, Faculty of Health Sciences, The University of Sydney / Institute for Neuroscience and Muscle Research / Paediatric Gait Analysis Service of NSW, Sydney Children’s Hospitals Network (Randwick and Westmead), Australia

## Background

Night-time calf muscle cramps are highly prevalent and painful, yet the underlying mechanism is poorly understood and no treatment has shown consistent efficacy or safety. The aim of this study was to identify factors associated with night-time calf cramping in adults.

## Methods

160 adults were recruited the Greater Newcastle and Central Coast regions of New South Wales, Australia: 80 who experienced night-time calf cramp at least once per week and 80 age- and sex-matched adults who did not. Participants were assessed using reliable tests of foot/ankle and toe strength, range of ankle dorsiflexion, hamstring flexibility, foot alignment, calf circumference, peripheral circulation and sensation. Participants also completed a bespoke survey examining health and lifestyle factors, diet, exercise, lower limb symptoms, sleeping habits and footwear characteristics.

## Results

Presence of night-time calf muscle cramps was significantly correlated with weakness of foot and ankle inversion, eversion, dorsiflexion and plantarflexion; weakness of toe grip; restricted hamstring flexibility; lower limb tingling sensations; muscle twitching, and coldness of legs or feet in bed at night. Conditional logistic regression identified three factors independently associated with night-time calf muscle cramps: muscle twitching (OR 4.6; 95%CI: 1.6 to 15.5; *p*=0.01), lower limb tingling (OR 4.1; 95%CI: 1.6 to 10.3; *p*=0.003) and foot dorsiflexion weakness (OR 1.02; 95%CI: 1.01 to 1.03; *p*=0.002), which represented other measures of lower limb weakness in the model.

## Conclusion

Night-time calf muscle cramps were associated with markers of neurological dysfunction and potential musculoskeletal therapeutic targets.

